# Identification of PI3K/AKT/mTOR-related genes as diagnostic biomarkers for cutaneous squamous cell carcinoma

**DOI:** 10.1016/j.bbrep.2025.102355

**Published:** 2025-12-02

**Authors:** Bingyan Yang, Hongyang Zhang, Lingdi Dong, Jianjun Wang, Nan Yu

**Affiliations:** Department of Dermatology, General Hospital of Ningxia Medical University, Yinchuan, Ningxia, China

**Keywords:** Cutaneous squamous cell carcinoma, PI3K/AKT/mTOR pathway, Bioinformatic analysis, Therapeutic target

## Abstract

**Background:**

Cutaneous squamous cell carcinoma (cSCC) is a common type of skin cancer. Considering the substantial improvement in prognosis when detected at an early stage, identifying biomarkers for an early diagnosis of cSCC is crucial. The phosphoinositide 3-kinase (PI3K)/protein kinase B (AKT)/mammalian target of rapamycin (mTOR) pathway plays a crucial role in cSCC progression; This study aimed to identify PI3K/AKT/mTOR-related genes that may serve as diagnostic indicators for cSCC, thereby providing a diagnostic framework for this disease.

**Methods/results:**

A total of 33 PI3K/AKT/mTOR-related differentially expressed genes (DEGs) in cSCC were acquired by intersecting the DEGs from the Gene Expression Omnibus database between normal and cSCC groups and genes reported to be associated with the PI3K/AKT/mTOR pathway in the literature. LASSO regression identified 11 hub genes (*AKT1, AKT3, EIF4EBP1, GFRA1, GRSF1, HIF1A, IGF1, IL11, IL24, KRT75,* and *MMP3*), which were used to construct the diagnostic model. Receiver operating curve analysis revealed that these hub genes displayed strong diagnostic capacity. Quantitative polymerase chain reaction validation confirmed significant differences in mRNA expression of *HIF1A, MMP3, IL11, GRSF1,* and *EIF4EBP1* between cSCC and normal cell lines.

**Conclusion:**

These five PI3K/AKT/mTOR-related genes have the potential to serve as clinical biomarkers for the diagnosis of cSCC and as candidate therapeutic targets. This study offers valuable insights for further research to elucidate the specific pathological mechanisms and establish innovative treatment approaches for cSCC.

## Highlights

• Dentifies a novel diagnostic model for cutaneous squamous cell carcinoma (cSCC) using 11 PI3K/AKT/mTOR pathway hub genes.
• Validates HIF1A, MMP3, IL11, GRSF1, and EIF4EBP1 as differentially expressed PI3K/AKT/mTOR-related genes in cSCC cell lines.
• 3. Proposes HIF1A, MMP3, IL11, GRSF1, and EIF4EBP1 as potential clinical diagnostic biomarkers and therapeutic targets for cSCC.

## 1 Introduction

Cutaneous squamous cell carcinoma (cSCC) is a malignancy resulting from the keratinocytes of the epidermis or epidermal appendages [1]. A cSCC is the most common non-melanoma skin cancer and accounts for about 20 % of all cutaneous cancers, second only to basal cell carcinomas [2]. The incidence rates of cSCC are increasing globally and are predicted to continue to rise [2]. Although the majority of individuals diagnosed with cSCC have positive prognoses, metastasis occurs in approximately 1.5–5.2 % of cases. Approximately 90 % of cSCC cases can be effectively treated with surgery; however, 5 % progress to advanced stages that are not suitable for radical surgery or radiotherapy owing to the complexities of extensive lesions, bone erosion, and deeper tumor invasion, typically accompanied by multiple recurrences [3]. Advanced cSCC represents a serious threat to human skin and overall health. Therefore, the early diagnosis and prevention of cSCC are crucial. Currently, cSCC diagnosis mainly relies on clinical features, dermatoscopy findings, skin biopsy, and histopathology. Given the intricate biological nature of cSCC, the clinical presentation of atypical or challenging cases, particularly in early or precancerous lesions, necessitates further evidence to aid in risk stratification and quantification of lesion progression potential [2]. Consequently, a diagnostic model and biomarkers that can effectively address these deficiencies in early diagnosis are urgently required. The phosphoinositide 3-kinase (PI3K)/protein kinase B (AKT)/mammalian target of rapamycin (mTOR) pathway is a classical signaling pathway [4] that plays a crucial role in cSCC progression [5]. The loss of phosphatase and tensin homolog causes the activation of the PI3K/AKT/mTOR signaling pathway, which in turn affects the expression of the human epidermal growth factor receptor (EGFR), resulting in the promotion of cSCC cell proliferation, migration, and invasion [6]. Moreover, EGFR expression is affected by ultraviolet radiation, leading to an overactive PI3K/AKT/mTOR pathway in cSCC. Metastatic cSCC is characterized by a significant presence of phosphorylated mTOR, further indicating the involvement of this pathway in the advancement of cSCC [7]. These discoveries highlight the significance of the PI3K/AKT/mTOR pathway in the growth and progression of cSCC by facilitating cell proliferation and hindering apoptosis. Further research on the specific roles of components in the PI3Ks/Akt/mTOR pathway is crucial to identify effective molecular targets and treatment strategies for cSCC.Therefore, comprehensive analysis of differentially expressed PI3K/AKT/mTOR-related genes may serve as potential diagnostic markers for cSCC, and corresponding diagnostic models could effectively guide clinical decisions.

**Fig. 1:**
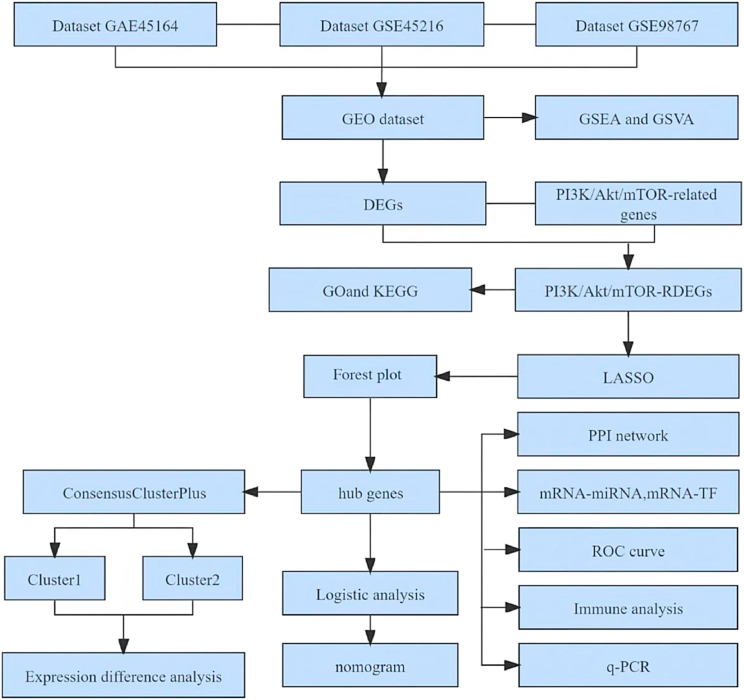
Workflow of this study. cSCC, cutaneous squamous cell carcinoma; GSEA: gene set enrichment analysis; GSVA: gene set variation analysis; GO: Gene Ontology; KEGG: Kyoto Encyclopedia of Genes and Genomes; PPI: protein–protein interaction; LASSO: least absolute shrinkage and selection operator; ROC: receiver operating characteristic; PI3K/Akt/mTOR-RDEGs: PI3K/Akt/mTOR-related differentially expressed genes; DEGs: differentially expressed genes.

To establish a diagnostic model, we analyzed different public datasets of cSCC from the Gene Expression Omnibus (GEO) database from various perspectives. We used differential analysis, enrichment analysis, immune infiltration analysis, and machine-learning methods (LASSO regression) to identify candidate diagnostic markers. Subsequently, logistic regression and receiver operating curve (ROC) analyses were used to construct and assess the performance of the diagnostic model, respectively. Finally, the mRNA expression of the hub genes used to build the cSCC cell was verified in cSCC and normal cell lines. The workflow of the study design is schematically outlined in Fig. 1. The findings of this study are clinically significant, enriching the pool of candidate genes to improve the early diagnosis of cSCC and gain further insight into its pathogenesis.

## 2 Materials and methods

### 2.1 Data source and processing

**Fig. 2:**
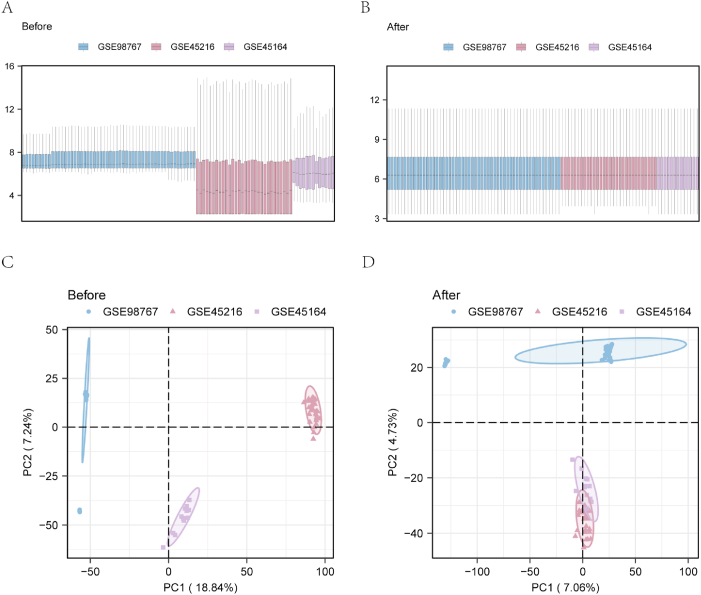
GEO datasets of sSCC. (**a-b**) Boxplot diagrams before (**a**) and after (**b**) batch effects were removed. **(c**–**d)** PCA plots before (**c**) and after (**d**) batch effects were removed. GEO: Gene Expression Omnibus; cSCC: cutaneous squamous cell carcinoma; PCA:Principal Component Analysis. Blue is for GSE98767, red is for GSE45216, and purple is for GSE45164.

The datasets for cSCC (GSE45164 [8], GSE45216 [9], and GSE98767 [10]) were acquired using GEO query software and retrieved from the GEO database [11] (http://www.ncbi.nlm.nih.gov/geo). The datasets only for *Homo sapiens* were obtained. The GPL571HG-U133A_2 Affymetrix Human Genome U133A 2.0 array served as the detection platform for the GSE45164 dataset with the obtained gene expression profiles for cSCC (n = 10) and normal (n = 3) tissues. The GPL570HG-U133_Plus_2 Affymetrix Human Genome U133 Plus 2.0 array served as the detection platform for the GSE45216 dataset, with gene expression profiles for only cSCC tissues (n = 30). The GSE98767 dataset was obtained using a GPL10558 Illumina HumanHT-12 V4.0 expression bead chip as the detection platform, including gene expression profiles for cSCC (n = 45) and normal (n = 9) tissues. The detailed information of these datasets is provided in Table 1. We identified 117 PI3K/AKT/mTOR-related genes from the literature [12] after de-duplication of the data (see Supplementary Table S1 online). To improve statistical power owing to the limited sample size of each dataset,the GSE45164, GSE45216, and GSE98767 GEO datasets were merged after implementing uniform gene annotation standards, and batch effects for samples from different sources were eliminated using the R package “sva” (Fig. 2a and b). The combined dataset was standardized with the “limma” package. The combined GEO dataset included a total of 85 cSCC and 12 samples of normal controls.Identification of PI3K/AKT/mTOR-related differentially expressed genes (DEGs) in cSCC. We acquired DEGs between different groups (normal and cSCC) by performing differential expression analysis on the combined GEO dataset using the “limma” software package^12^. We considered genes with a |log fold change (FC)| > 0 and P < 0.05 as significant DEGs for further study. Genes with a log FC > 0 and P < 0.05 were considered upregulated genes, whereas those with a log FC < 0 and P < 0.05 were considered downregulated genes in cSCC.

**Table 1:** Cutaneous squamous cell carcinoma (cSCC) Gene Expression Omnibus (GEO) dataset information.

	GSE45164	GSE45216	GSE98767
Platform	GPL571	GPL570	GPL10558
Species	Homo sapiens	Homo sapiens	Homo sapiens
Samples in Normal group	3		9
Samples in cSCC group	10	30	45
Reference	8	9	10

The PI3K/AKT/mTOR-related DEGs associated with cSCC were identified by the intersection of DEGs in the GEO dataset and the PI3K/AKT/mTOR-related genes reported in the literature [13], using a Venn diagram. A volcano plot of the DEGs was constructed using the R package “ggplot.”

### 2.2 Enrichment analyses

#### 2.2.1 Functional enrichment analysis of PI3K/AKT/mTOR-related DEGs

Gene Ontology (GO) was used for functional enrichment, encompassing biological process (BP), molecular function (MF), and cellular component (CC) terms [14]. Kyoto Encyclopedia of Genes and Genomes (KEGG) [15] was used to determine the pathways associated with the identified DEGs, which provides extensive data on genomes, pathways, diseases, and drugs. The R program “clusterProfiler” was used for enrichment analysis [16]. The enrichment set was considered significant if the P value was <0.05 and the false discovery rate (FDR; q value) was <0.05. The P value was adjusted using the Benjamini–Hochberg method.

#### 2.2.2 Gene set enrichment analysis (GSEA) and gene set variation analysis (GSVA) of the cSCC dataset

Gene distribution trends were evaluated using GSEA, referring to phenotypic correlation-sequenced gene lists [17], thereby determining their contribution to phenotypes. Using clusterProfiler, we ran GSEA on all genes associated with phenotypes, with the gene list pre-ranked according to log FC values to assess whether there was significant enrichment in a predefined set of genes. GSEA parameters were as follows: seeds = 2020, repeats = 1000, and genes per gene set = 10–500. Based on the Molecular Signatures Database (MSigDB), we used the “hall.v7.4. symbols.gmt” gene set to analyze the GEO data. The threshold value for statistical significance in GSEA was set to P < 0.05 and FDR (q value) < 0.25. The P value was corrected using the Benjamini–Hochberg method.

GSVA is an unsupervised, non-parametric technique for gene set analysis [18]. Gene enrichment in microarrays was assessed through this analysis, and transcripts in the gene expression matrix were converted to gene sets to evaluate whether different pathways were enriched in different samples. The “h.all.v7.4.symbols.gmt” gene set was collected from the GEO MSigDB database, and functional enrichment differences between the datasets of normal and cSCC groups were evaluated.

### 2.3 Construction of the PI3K/AKT/mTOR-related DEG diagnostic model

We constructed the diagnostic model for cSCC using LASSO regression with “binomial” parameters in the “glmnet” package [19] to identify hub genes among the PI3K/AKT/mTOR-related DEGs; 10-fold cross-validation with 1000 cycles was used to avoid overfitting. LASSO regression is a popular machine-learning approach for constructing diagnostic models that employs linear regression and further incorporates regularization and a penalty term (the lambda absolute value of the slope) to address the overfitting issue. Using the diagnostic model from LASSO regression, we computed the risk scores linked to DEGs related to the PI3K/AKT/mTOR pathway based on the following formula:

$$\text{riskScore}=\sum_{i}\text{Coefficient}\;\left({\text{hub}\;\text{gene}}_{i}\right)\times \text{mRNA}\;\text{Expression}\;\left(\text{hub}\;{\text{gene}}_{i}\right)$$

The median risk score was employed to categorize all cSCC samples in the GEO dataset into groups according to high and low risk. The R “Circos” package was used to annotate the positions of PI3K/AKT/mTOR-related DEGs on human chromosomes.

### 2.4 Immune infiltration analysis

The abundance profiles of 22 subtypes of immune cells were used to assess the condition of the immune cells infiltrating the samples in the GEO dataset, which were assessed using the CIBERSORT tool (https://cibersort.stanford.edu/) [20] by deconvoluting immune cell subtype expression matrices using linear support vector regression. Spearman's correlation analysis was employed to examine the correlations between hub genes and immune-infiltrating cells. Using the cSCC dataset gene expression matrix, we further examined the relationships between immune cells and PI3K/AKT/mTOR-related DEGs. Finally, a heatmap depicting the gene–immune cell correlations was plotted using the “heatmap” R package.

### 2.5 Identification of disease different subtypes or risk groups of cSCC

The GEO dataset was used to identify different subtypes or risk groups of cSCC using the consensus clustering method [21] for identification of each sample and its corresponding subgroup, followed by the validation of the cluster to ensure its rationality. The R package “ConsensusClusterPlus” [22] was used to cluster the filtered DEGs related to the PI3K/AKT/mTOR pathway. The range of clusters was established as 2–8, with 50 repetitions for 80 % of the overall samples. The clusterAlg parameter was configured as “km” with the distance type specified as “Euclidean.” The Mann–Whitney *U* test (Wilcoxon rank-sum test) was employed to confirm the expression of PI3K/AKT/mTOR-related DEGs across various cSCC subtypes. P < 0.05 was considered statistically significant.

### 2.6 Construction of protein–protein interaction (PPI), mRNA–microRNA (miRNA), and mRNA–RNA-binding protein (RBP) networks

The molecular mechanisms underlying cSCC were revealed using the STRING (http://string-db.org) database [23] to retrieve interactions between known and predicted proteins. We constructed a PPI network for the screened PI3K/AKT/mTOR-related DEGs, which was visualized using Cytoscape. ENCORI [24] (http://starbase.sysu.edu.cn/) version 3.0 of the starBase database contains information on miRNA–non-coding RNA (ncRNA), miRNA–mRNA, ncRNA–RNA, RNA–RNA, RBP–ncRNA, and RBP–mRNA interactions, offering various visual interfaces for investigating miRNA targets. We used the miRDB database to predict miRNA target genes and annotate their functions [25]. The miRNA–miRNA and mRNA–RBP interaction networks were constructed using the ENCORI [24] (https://starbase.sysu.edu.cn/) and miRDB databases.

### 2.7 Performance evaluation of the diagnostic model

The “pROC” package was used to generate ROC curves for hub genes in the GEO dataset, offering an aggregate indicator that demonstrates the connection between sensitivity and specificity. The effect of hub gene expression on the disease was evaluated using the area under the ROC curve (AUC) value, which varies from 0.5 to 1; the closer the AUC is to 1, the better the prediction.

### 2.8 Validation of hub gene expression differences in vitro

The cSCC cell line A431 (iCell-h009, RRID:CVCL_0037) and the normal human keratinocyte cell line HaCat (iCell-h066, RRID:CVCL_0038) (both purchased from iCell Bioscience Inc., Shanghai, China) were used to validate the expressions of the screened hub genes from the GEO database using reverse transcription-quantitative polymerase chain reaction (RT-qPCR). The cells were cultured in Dulbecco's modified Eagle medium (KGM12800S, KeyGEN Bio Tech) and 10 % fetal bovine serum without antibiotics in a 5 % CO_2_ incubator at 37 °C. The medium was changed every 2–3 days.

Total RNA was extracted from A431 and HaCat cells using the CWBIO UItrapure RNA kit (CW0581 M). The reverse transcription of total RNA into cDNA was performed using HiScript® II Q RT SuperMix for qPCR (+gDNA wiper) (R223-01, Vazyme biotech.com). The reverse transcription process involved incubation at 50 °C for 15 min, followed by incubation at 85 °C for 5 s. For qPCR, the thermocycling conditions were as follows: an initial step at 95 °C for 10 min, followed by 40 cycles at 95 °C for 10 s, 58 °C for 30 s, and an extension at 72 °C for 30 s. The 2ΔΔCq method was used to determine the relative gene expression. The primers used for qPCR are provided in Supplementary Table S2 online. We normalized the expression of target mRNAs to the internal reference gene, β-actin.This experiment was conducted to validate the expression levels of selected key genes, thereby reinforcing the reliability of our findings.

### 2.9 Statistical analysis

R software (version 4.1.2) was used to process and analyze all data. For normally distributed continuous variables between groups, an independent Student *t*-test was employed to assess statistical significance, whereas for non-normally distributed continuous variables, the Mann–Whitney *U* test was employed. To assess and analyze the statistical significance between two groups of categorical variables, either the Chi-square test or Fisher's exact test was employed. In the absence of any other specifications, Spearman's correlation analysis was employed to calculate the correlation coefficients. P < 0.05 was considered statistically significant for all two-sided tests.

## 3 Results

### 3.1 Analysis of DEGs associated with cSCC

**Fig. 3:**
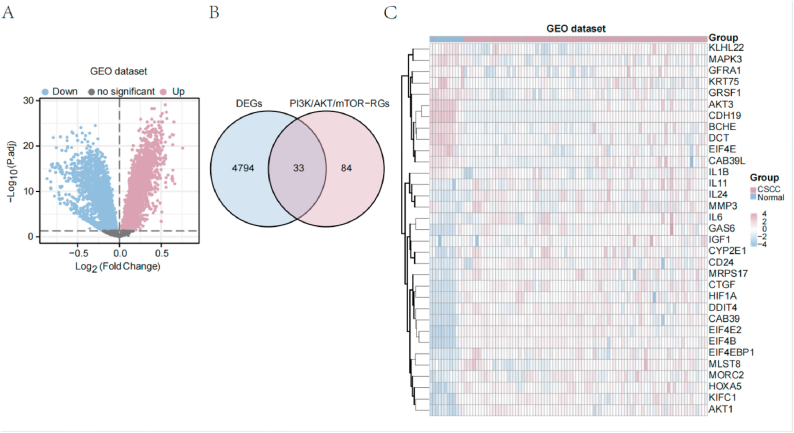
Analysis of DEGs in cSCC. (**a**) Volcano plot of DEGs in GEO datasets. (**b**) Venn diagrams between the GEO dataset DEGs and PI3K/Akt/mTOR-RGs. (**c**) Heatmap of DEGs in the GEO dataset. DEGs: differentially expressed genes; PI3K/Akt/mTOR-RGs: PI3K/Akt/mTOR-related genes.

A total of 4827 DEGs satisfied the criteria of |log FC| > 0 and P < 0.05 between cSCC and normal tissue samples, including 2618 upregulated genes (logFC >0) and 2209 downregulated genes (logFC <0) in the cSCC group, based on gene expression analysis using the “limma” package for the combined GEO dataset. Fig. 3a displays the final DEGs obtained through volcano maps. We obtained 33 PI3K/AKT/mTOR-related DEGs from the intersection of these DEGs identified in the GEO datasets with PI3K/AKT/mTOR-related genes extracted from the literature using a Venn diagram (Fig. 3b). The expression levels of the 33 PI3K/AKT/mTOR-related DEGs are presented in a heatmap in Fig. 3c, showing significant clustering for the two groups.

### 3.2 Functional enrichment analysis of PI3K/AKT/mTOR-related DEGs

**Fig. 4:**
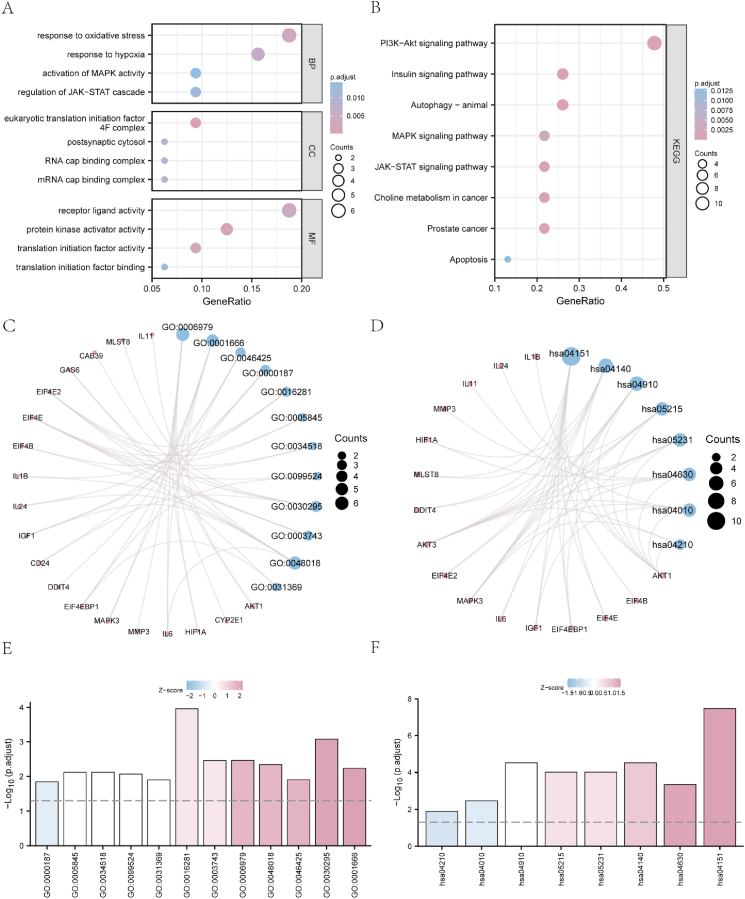
GO and KEGG enrichment analyses of PI3K/Akt/mTOR-RDEGs. (**a-b**) Bubble diagram of GO (**a**) and KEGG pathway (**b**) enrichment analysis of PI3K/Akt/mTOR-RDEGs. (c–d) Ring network chart of GO (**c**) and KEGG pathway (**d**) functional enrichment analysis of PI3K/Akt/mTOR-RDEGs. **(e-f**) Bar graph of GO (**e**) and KEGG pathway (**f**) functional enrichment analysis of PI3K/Akt/mTOR-RDEGs. PI3K/Akt/mTOR-RDEGs: PI3K/Akt/mTOR-related differentially expressed genes; GO: Gene Ontology; BP: biological process; CC: cellular component; MF: molecular function; KEGG: Kyoto Encyclopedia of Genes and Genomes. The screening criteria of enriched terms were P value < 0.05 and false discovery rate (FDR; q value) < 0.05.

Table 2, Table 3 display the results of GO and KEGG pathway enrichment for the 33 PI3K/AKT/mTOR-related DEGs, respectively. These results are visualized as bubble diagrams in Fig. 4a and b, and as ring network charts in Fig. 4c and d. As shown in Fig. 4e and f, these 33 DEGs were primarily enriched in BP terms such as mitogen-activated protein kinase (MAPK) activity (GO 0000187), regulation of the JAK–STAT cascade (GO 0046425), response to hypoxia (GO 0001666), and response to oxidative stress (GO 0006979); CC terms such as eukaryotic translation initiation factor 4F complex (eIF4F; GO 0016281), mRNA cap-binding complex (GO 0005845), RNA cap-binding complex (GO 0034518), and postsynaptic cytosol (GO 0099524); and MF terms such as protein kinase activator activity (GO 0030295), translation initiation factor activity (GO 0003743), receptor-ligand activity (GO 0048018), and translation initiation factor binding (GO 0031369). Enrichment of the 33 PI3K/AKT/mTOR-related DEGs was primarily observed in KEGG pathways such as PI3K/AKT (hsa04151), MAPK (hsa04010), JAK–STAT (hsa04630), autophagy-animal (hsa04140), apoptosis (hsa04210), insulin signaling (hsa04910), prostate cancer (hsa05215), and choline metabolism in cancer (hsa05231).

**Table 2:** GO enrichment analysis of PI3K/Akt/mTOR-related differentially expressed genes.

ONTOLOGY	ID	Description	GeneRatio	BgRatio	P value	P adjust	q value
BP	GO:0031929	TOR signaling	8/32	125/18670	2.96e-11	5.78e-08	2.83e-08
BP	GO:0018105	peptidyl-serine phosphorylation	9/32	299/18670	1.25e-09	1.22e-06	5.97e-07
BP	GO:0018209	peptidyl-serine modification	9/32	322/18670	2.39e-09	1.56e-06	7.62e-07
BP	GO:0032006	regulation of TOR signaling	6/32	105/18670	2.20e-08	1.08e-05	5.27e-06
BP	GO:0035690	cellular response to drug	8/32	369/18670	1.50e-07	5.86e-05	2.87e-05
CC	GO:0016281	eukaryotic translation initiation factor 4F complex	3/32	13/19717	1.10e-06	1.10e-04	8.56e-05
CC	GO:0005845	mRNA cap binding complex	2/32	12/19717	1.67e-04	0.008	0.006
CC	GO:0034518	RNA cap binding complex	2/32	14/19717	2.29e-04	0.008	0.006
CC	GO:0099524	postsynaptic cytosol	2/32	17/19717	3.42e-04	0.009	0.007
CC	GO:0099522	region of cytosol	2/32	24/19717	6.89e-04	0.014	0.011
MF	GO:0030295	protein kinase activator activity	4/32	80/17697	1.26e-05	8.36e-04	6.20e-04
MF	GO:0019209	kinase activator activity	4/32	86/17697	1.68e-05	8.36e-04	6.20e-04
MF	GO:0071889	14-3-3 protein binding	3/32	29/17697	1.90e-05	8.36e-04	6.20e-04
MF	GO:0003743	translation initiation factor activity	3/32	51/17697	1.05e-04	0.003	0.003
MF	GO:0048018	receptor ligand activity	6/32	482/17697	1.96e-04	0.005	0.003

GO: Gene Ontology; BP: biological process; CC: cellular component; MF: molecular function.

**Table 3:** KEGG enrichment analysis of PI3K/Akt/mTOR-related differentially expressed genes.

ONTOLOGY	ID	Description	GeneRatio	BgRatio	P value	P adjust	q value
KEGG	hsa04150	mTOR signaling pathway	12/23	155/8076	1.84e-15	2.99e-13	8.89e-14
KEGG	hsa01521	EGFR tyrosine kinase inhibitor resistance	9/23	79/8076	3.76e-13	3.06e-11	9.10e-12
KEGG	hsa04066	HIF-1 signaling pathway	9/23	109/8076	7.43e-12	4.04e-10	1.20e-10
KEGG	hsa04151	PI3K-Akt signaling pathway	11/23	354/8076	8.29e-10	3.38e-08	1.00e-08
KEGG	hsa04211	Longevity regulating pathway	6/23	89/8076	1.31e-07	4.28e-06	1.27e-06

KEGG: Kyoto Encyclopedia of Genes and Genomes.

### 3.3 GSEA and GSVA of the cSCC dataset

**Fig. 5:**
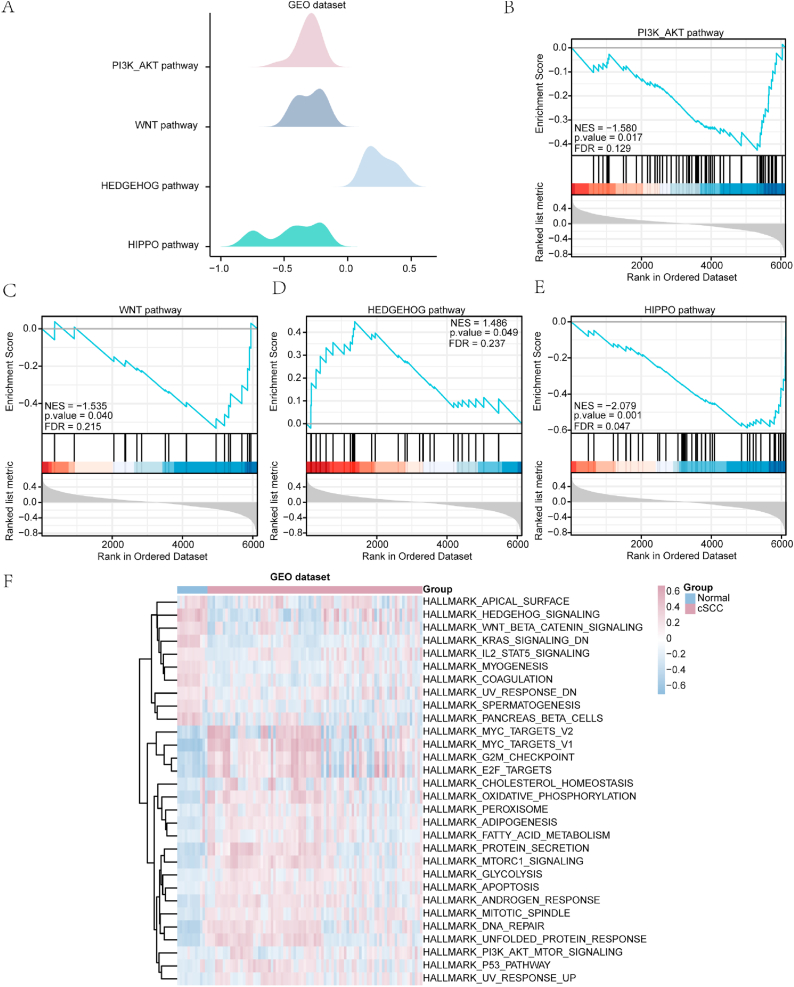
GSEA and GSVA of PI3K/Akt/mTOR-related genes in the GEO dataset. (**a**) Ridge plot of GSEA of the four major biological characteristics. (**b–e**) Significant enrichment in the PI3K/AKT pathway (**b**), WNT pathway (**c**), Hedgehog pathway (**d**), and Hippo pathway (**e**). (**f**) GSVA in the GEO dataset. Blue represents the normal group and red represents the cSCC group. cSCC: cutaneous squamous cell carcinoma; GSEA: gene set enrichment analysis. GSVA: gene set variation analysis. The screening criteria for significantly enriched terms were P value < 0.05 and false discovery rate (FDR; q value) < 0.05.

The effects of PI3K/AKT/mTOR-related gene expression levels on cSCC were evaluated using GSEA according to the relationships between GO enrichment categories (BP, CC, and MF) and gene expression levels in the normal and cSCC groups. The GSEA results were visualized using a ridge plot (Fig. 5a). The PI3K/AKT (Fig. 5b), WNT (Fig. 5c), Hedgehog (Fig. 5d), Hippo (Fig. 5e), and various other signaling pathways (Table 4) exhibited significant enrichment of all genes within the GEO dataset.

**Table 4:** Gene set enrichment analysis of the Gene Expression Omnibus dataset for cutaneous squamous cell carcinoma.

Description	setSize	enrichmentScore	NES	P value	P adjust	q values
REACTOME_NEGATIVE_REGULATION_OF_THE_PI3K_AKT_NETWORK	61	−0.425968502	−1.579582219	0.017123288	0.148917954	0.12856847
WP_WNT_SIGNALING_IN_KIDNEY_DISEASE	18	−0.532376756	−1.534811823	0.040145985	0.249488367	0.215396041
PID_HEDGEHOG_GLI_PATHWAY	28	0.446745204	1.486226088	0.049217002	0.274064451	0.236613828
WP_PATHWAYS_REGULATING_HIPPO_SIGNALING	46	−0.587286858	−2.079455357	0.001718213	0.054286473	0.046868283
REACTOME_NEGATIVE_REGULATION_OF_THE_PI3K_AKT_NETWORK	61	−0.425968502	−1.579582219	0.017123288	0.148917954	0.12856847
NABA_MATRISOME	494	−0.382509873	−1.890708528	0.001416431	0.054286473	0.046868283
REACTOME_SIGNALING_BY_GPCR	372	−0.344825913	−1.666030325	0.001488095	0.054286473	0.046868283
NABA_MATRISOME_ASSOCIATED	361	−0.333254646	−1.606168431	0.001497006	0.054286473	0.046868283
REACTOME_EXTRACELLULAR_MATRIX_ORGANIZATION	205	−0.389840155	−1.765797955	0.001517451	0.054286473	0.046868283
REACTOME_GPCR_LIGAND_BINDING	230	−0.423582501	−1.939748981	0.001531394	0.054286473	0.046868283
KEGG_NEUROACTIVE_LIGAND_RECEPTOR_INTERACTION	170	−0.541761193	−2.396600244	0.001569859	0.054286473	0.046868283
REACTOME_CLASS_A_1_RHODOPSIN_LIKE_RECEPTORS_	175	−0.414238667	−1.833689603	0.001569859	0.054286473	0.046868283
REACTOME_TRANSMISSION_ACROSS_CHEMICAL_SYNAPSES	150	−0.385705836	−1.672216724	0.001582278	0.054286473	0.046868283
WP_GPCRS_CLASS_A_RHODOPSINLIKE	132	−0.438973325	−1.861384285	0.001633987	0.054286473	0.046868283
NABA_CORE_MATRISOME	133	−0.496476309	−2.102744211	0.001650165	0.054286473	0.046868283

We further performed GSVA (Fig. 5f) between the cSCC samples and their normal counterparts in the GEO datasets to explore the differential functional enrichment of the two groups. The differentially enriched gene sets of pathways included the Hedgehog, WNT, and interleukin (IL)-2 pathways (Table 5).

**Table 5:** Gene set variation analysis of the Gene Expression Omnibus dataset for cutaneous squamous cell carcinoma.

	logFC	AveExpr	t	P value	adj P Value
HALLMARK_KRAS_SIGNALING_DN	0.463794502	−0.099662597	8.267623652	2.34E-13	1.17E-11
HALLMARK_MYC_TARGETS_V1	−0.649156724	0.059657502	−6.692809418	7.78E-10	1.95E-08
HALLMARK_PROTEIN_SECRETION	−0.492214854	0.047401276	−6.379410693	3.62E-09	6.03E-08
HALLMARK_G2M_CHECKPOINT	−0.533374952	0.063563067	−5.920617818	3.23E-08	4.04E-07
HALLMARK_DNA_REPAIR	−0.456671787	0.06405626	−5.786661673	6.03E-08	6.03E-07
HALLMARK_MTORC1_SIGNALING	−0.416131285	0.057275716	−5.552329296	1.77E-07	1.47E-06
HALLMARK_COAGULATION	0.359921179	−0.056265911	5.376158857	3.90E-07	2.72E-06
HALLMARK_E2F_TARGETS	−0.542361306	0.054021706	−5.351717914	4.35E-07	2.72E-06
HALLMARK_UNFOLDED_PROTEIN_RESPONSE	−0.417013656	0.034433879	−5.229351299	7.46E-07	4.15E-06
HALLMARK_MITOTIC_SPINDLE	−0.328271958	0.046412357	−5.019532305	1.85E-06	9.27E-06
HALLMARK_HEDGEHOG_SIGNALING	0.483934155	−0.012131502	4.963353999	2.36E-06	9.97E-06
HALLMARK_MYOGENESIS	0.328086864	−0.029204237	4.959873337	2.39E-06	9.97E-06
HALLMARK_ADIPOGENESIS	−0.313969312	0.040349092	−4.703831228	7.00E-06	2.69E-05
HALLMARK_PANCREAS_BETA_CELLS	0.367158037	−0.060469228	4.487140953	1.69E-05	6.03E-05
HALLMARK_ANDROGEN_RESPONSE	−0.317762554	0.046717097	−4.430066217	2.12E-05	7.07E-05

### 3.4 Construction of a PI3K/AKT/mTOR-related DEG diagnostic model

**Fig. 6:**
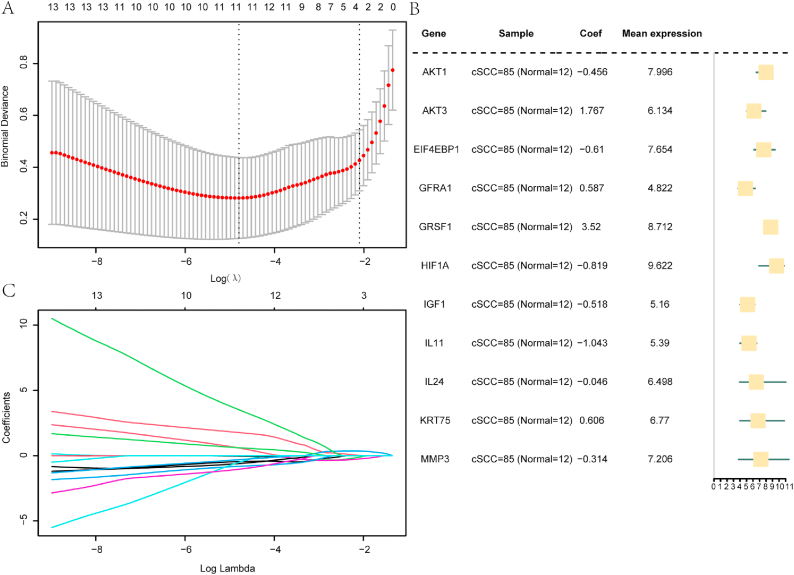
Construction of the PI3K/Akt/mTOR-RDEG diagnostic model. (**a**) LASSO regression diagnostic model diagram of PI3K/Akt/mTOR-RDEGs in the GEO dataset. (**b**) Forest plot of PI3K/Akt/mTOR-RDEGs in the diagnostic model. (**c**) LASSO variable trajectory map of the PI3K/Akt/mTOR-RDEGs diagnostic model. PI3K/Akt/mTOR-RDEGs: PI3K/Akt/mTOR-related differentially expressed genes; LASSO: least absolute shrinkage and selection operator.

We first constructed a diagnostic model based on the 33 PI3K/AKT/mTOR-related DEGs (Fig. 6a) using LASSO regression analysis. As shown in the forest plots in Figs. 6b and 11 of the 33 DEGs (*AKT1, AKT3, EIF4EBP1, GFRA1, GRSF1, HIF1A, IGF1, IL11, IL24, KRT75,* and *MMP3*) were ultimately selected for inclusion in the diagnostic model. The LASSO variable trajectory map is shown in Fig. 6c; as lambda coefficients decrease to 0, the number of genes gradually increases, demonstrating that genes change when LASSO penalty coefficients are implemented (lambda after log transformation).

**Fig. 7:**
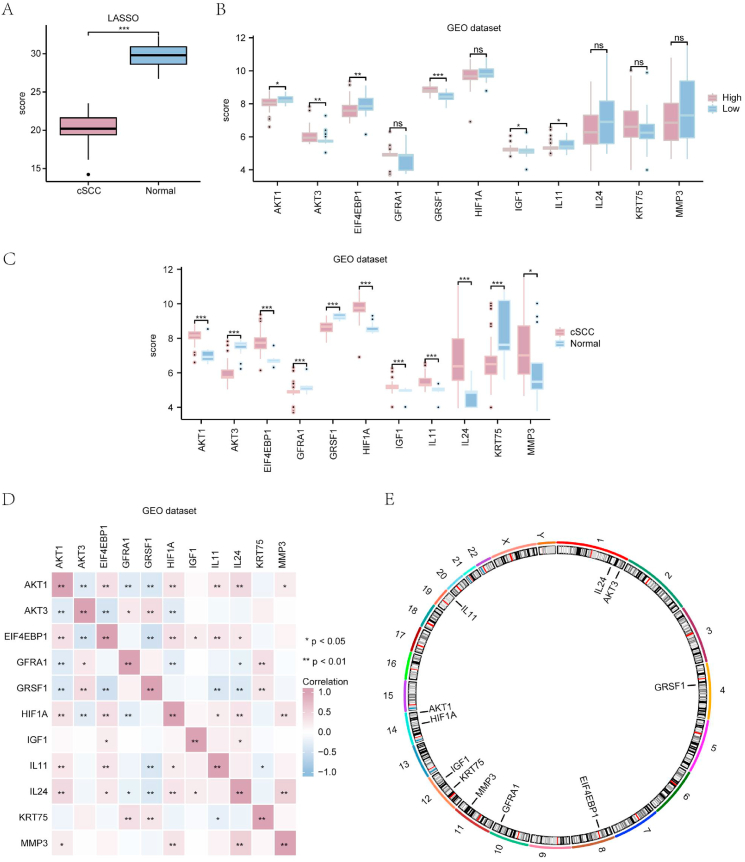
Expression of PI3K/Akt/mTOR-RDEGs in the cSCC GEO dataset. (**a**) Predictive scores for the diagnostic model based on differences in expression levels of PI3K/Akt/mTOR-RDEGs between cSCC (red) and normal (blue) groups. (**b**) Expression levels of the PI3K/Akt/mTOR-RDEGs in the diagnostic model according to high-risk (red) and low-risk (blue) groups in the cSCC samples. (**c**) Expression level of PI3K/Akt/mTOR-RDEGs in different groups (cSCC and normal). (**d**) Correlation heat map of the PI3K/Akt/mTOR-RDEGs in the GEO dataset. ns, not significant (P ≥ 0.05); ∗P < 0.05, ∗∗P < 0.01; ∗∗∗P < 0.001.(**e**) Chromosome location map of PI3K/Akt/mTOR-RDEGs.

We next computed predictive scores for the diagnostic model using LASSO regression analysis to determine the coefficients of the 11 PI3K/AKT/mTOR-related DEGs. These scores were subsequently multiplied by the gene expression level of each sample. The Wilcoxon signed-rank test indicated a significant difference (P < 0.001) in the expression of these PI3K/AKT/mTOR-related DEGs between the cSCC and normal groups in the GEO dataset (Fig. 7a).

The cSCC group samples were divided into high- and low-risk groups based on the final prediction scores of the 11 genes in the diagnostic model (Fig. 7b). The Wilcoxon signed-rank test indicated significant variations in *GRSF1* (P < 0.001), *AKT3*, and *EIF4EBP1* (P < 0.01), as well as *AKT1, IGF1*, and *IL11* (P < 0.05) expression, between the risk groups.

Finally, we analyzed the expression of the 11 PI3K/AKT/mTOR-related DEGs included in the LASSO model between different groups (normal/cSCC) in the GEO dataset (Fig. 7c). A significant variation was observed in the expression of 10 out of the 11 hub genes (*AKT1, AKT3, EIF4EBP1, GFRA1, GRSF1, HIF1A, IGF1, IL11, IL24,* and *KRT75*) between the groups (P < 0.001). The expression of the remaining DEG (*MMP3*) exhibited a moderate but statistically significant difference between the cSCC and normal groups (P < 0.05). Among these DEGs, *AKT1, EIF4EBP1, HIF1A, IGF1, IL11, IL24,* and *MMP3* were upregulated, whereas *AKT3,GFRA1, GRSF1,* and *GRSF1* were downregulated in cSCC.

The correlation heatmap (Fig. 7d) revealed significant associations between the expressions of *AKT1* and *AKT3, EIF4EBP1, GFRA1, GRSF1, HIF1A, IL11,* and *IL24* in the GEO dataset (P < 0.01). A moderate but significant correlation was also observed between *AKT1* and *MMP3* expression (P < 0.05). The circle plot in Fig. 7e shows that these 11 genes are located at specific positions on the chromosomes.

### 3.5 PPI, hub genes–miRNA, and hub genes–RBP interaction networks

A PPI network was constructed by considering interactions of the 11 hub genes (*AKT1, AKT3, EIF4EBP1, GFRA1, GRSF1, HIF1A, IGF1, IL11, IL24, KRT75*, and *MMP3*) with a minimum score of 0.400 (medium confidence) and removing the non-relevant genes (see Supplementary Fig. 1Sa). Based on the intersection of the ENCORI and miRDB databases and Cytoscape software results, an mRNA–miRNA interaction network (see Supplementary Fig. 1Sb) identified 8 hub genes (*AKT1, AKT3, EIF4EBP1, GFRA1, GRSF1, HIF1A, IGF1,* and *IL24*) and 92 miRNAs for a total of 102 pairs of mRNA–miRNA interaction relationships (see Supplementary Table S3 online). In addition, the RBP–hub gene network (see Supplementary Fig. 1Sc) comprised 23 RBP molecules and 8 hub genes (*AKT1, AKT3, EIF4EBP1, GFRA1, GRSF1, HIF1A, IGF1,* and *IL11*), resulting in a total of 89 mRNA–RBP interactions. Please refer to Supplementary Table S4 online for more details.

### 3.6 Identification of cSCC disease subtypes

**Fig. 8:**
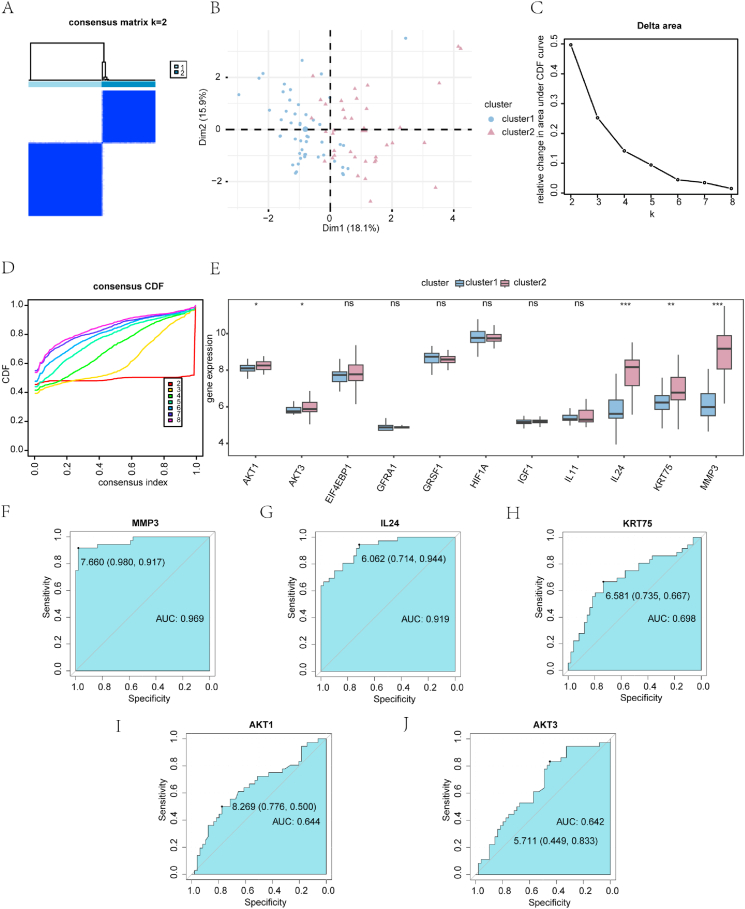
Determination of cSCC subtypes according to PI3K/Akt/mTOR-RDEGs. (**a**) Consistency clustering (k = 2) for cSCC in the GEO dataset. (**b**) Principal component analysis of two cSCC disease subtypes (cluster 1 and cluster 2). (**c-d**) Consensus clustering delta plot of CDF area under the curve for differing the number of clusters (**c**) and consensus clustering CDF plot (**d**). (**f–j**) ROC curves for the PI3K/Akt/mTOR-RDEGs *MMP3* (**f**), *IL24* (**g**), *KRT75* (**h**), *AKT1* (**i**), and *AKT3* (**j**) in discriminating subtypes of cSCC. The diagnostic efficacy improves as the AUC on the ROC curve is close to 1. AUC was interpreted as follows: 0.5–0.7, poor accuracy; 0.7–0.9, moderate accuracy; >0.9, high accuracy. ROC: receiver operating characteristic; CDF: cumulative distribution function; PI3K/Akt/mTOR-RDEGs: PI3K/Akt/mTOR-related differentially expressed genes; cSCC: cutaneous squamous cell carcinoma.

Based on the PI3K/AKT/mTOR-related DEGs, we identified two cSCC-related different subtypes or risk groups (clusters 1 and 2) (Fig. 8a) using consensus clustering with the R package “ConsensusClusterPlus”, containing 49 and 36 samples, respectively. Principal component analysis revealed significant differences between the two cSCC different subtypes or risk groups (Fig. 8b). The best clustering result was observed when two clusters were used for unsupervised clustering (k = 2), as indicated by the delta (Fig. 8c) and cumulative distribution function (CDF) plots (Fig. 8d) depicting the area under the CDF curve for varying numbers of clusters in the consistency clustering results. The Mann–Whitney *U* test revealed significant differences in the levels of *IL24*, *MMP3* (P < 0.001), *KRT75* (P < 0.01), *AKT1*, and *AKT3* (P < 0.05) between the two cSCC disease different subtypes or risk groups (clusters 1 and 2) in the GEO dataset (Fig. 8e). The ROC curves for PI3K/AKT/mTOR-related DEGs showed that the expression of *MMP3* (AUC = 0.969; Fig. 8f) and *IL24* (AUC = 0.919; Fig. 8g) had high accuracy in discriminating the two different subtypes or risk groups of cSCC in the GEO dataset, followed by *KRT75* (AUC = 0.698; Fig. 8h), *AKT1* (AUC = 0.644; Fig. 8i), and *AKT3* (AUC = 0.642; Fig. 8j).

### 3.7 Immune infiltration of cSCC

**Fig. 9:**
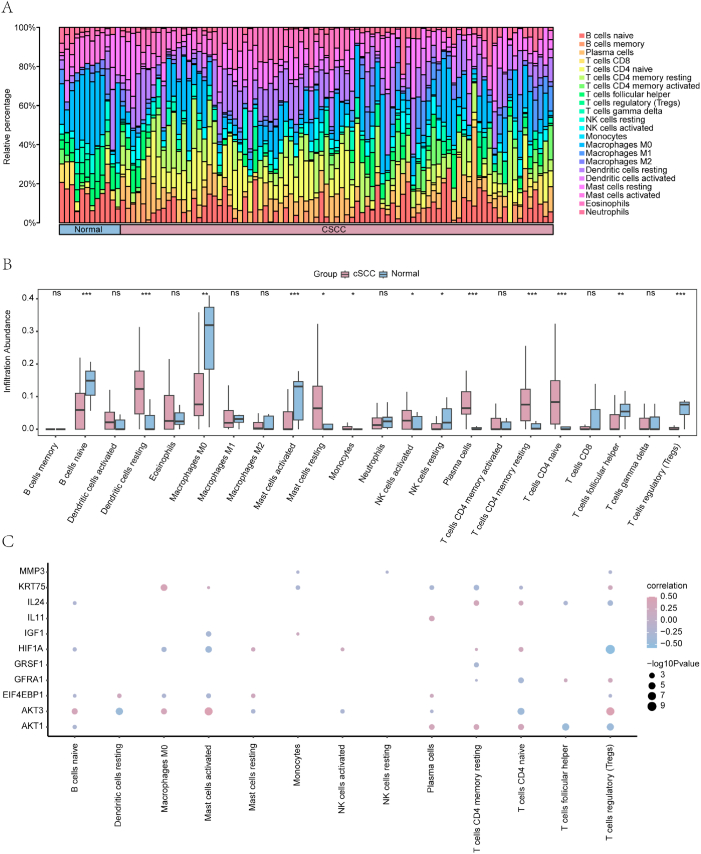
Immune infiltration analysis (CIBERSORT) of cSCC and normal samples in the GEO dataset. (**a**) Immune infiltration abundance for 22 immune cell types. (**b**) Comparison of immune cell infiltration abundance between different groups (normal/cSCC). (**c**) Heatmap depicting the correlations between PI3K/Akt/mTOR-RDEGs and immune cell abundance. Pink and blue circles demonstrate a positive and negative association between genes and immune cell infiltration abundance, respectively, with a larger circle representing a stronger correlation. ns, not significant (P ≥ 0.05); ∗P < 0.05; ∗∗P < 0.01; ∗∗∗P < 0.001. PI3K/Akt/mTOR-RDEGs: PI3K/Akt/mTOR-related differentially expressed genes; cSCC: cutaneous squamous cell carcinoma.

Fig. 9a and b displays notable variations (P < 0.001) in the levels of seven immune cells—inexperienced B cells, inactive dendritic cells, stimulated mast cells, plasma cells, dormant memory CD4^+^ T cells, inexperienced CD4^+^ T cells, and regulatory T cells (Tregs)—between cSCC and normal tissues. Moderately significant (P < 0.01) variations were also observed in the abundance of M0 macrophages and helper follicular T cells between the two groups. Additionally, four immune cell types (resting mast cells, monocytes, activated natural killer cells, and resting natural cells) exhibited significant differences between the two groups (P < 0.05). The associations between immune cell infiltration abundance and the expression of the 11 PI3K/AKT/mTOR-related DEGs are shown using correlation heat maps in Fig. 9c. In particular, a significant correlation was observed between mast cell abundance and *AKT3* expression.

### 3.8 Diagnostic value of hub genes in cSCC

**Fig. 10:**
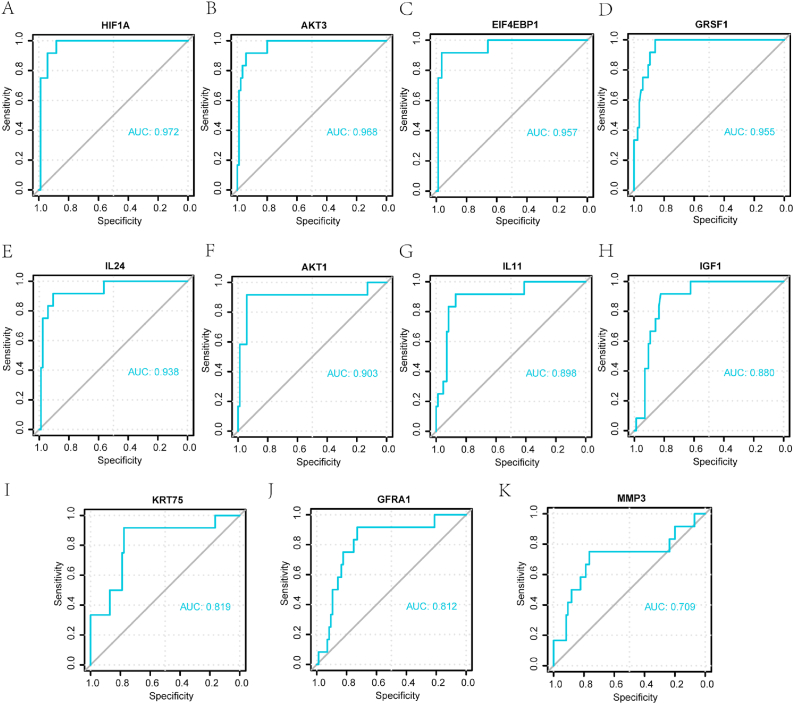
ROC curve analysis of the diagnostic value of hub genes for cSCC: *HIF1A* (**a**), *AKT3* (**b**), *EIF4EBP1* (**c**), *GRSF1* (**d**), *IL24* (**e**), *AKT1* (**f**), *IL11* (**g**), *IGF1* (**h**), *KRT75* (**i**), *GFRA1* (**j**), and *MMP3* (**k**). PI3K/Akt/mTOR-RDEGs: PI3K/Akt/mTOR-related differentially expressed genes; ROC: receiver operating characteristic. The diagnostic performance is considered to be better when the area under the curve (AUC) value is closer to 1, interpreted as follows: AUC = 0.5–0.7, poor accuracy; AUC = 0.7–0.9, moderate accuracy.

ROC analysis showed that the hub genes *HIF1A* (AUC = 0.972; Fig. 10a), *AKT3* (AUC = 0.968; Fig. 10b), *EIF4EBP1* (AUC = 0.957; Fig. 10c), *GRSF1* (AUC = 0.955; Fig. 10d), *IL24* (AUC = 0.938; Fig. 10e), and *AKT1* (AUC = 0.903; Fig. 10f) exhibited high diagnostic values between normal and cSCC samples based on their expression. The hub genes *IL11* (AUC = 0.898; Fig. 10g), *IGF1* (AUC = 0.880; Fig. 10h), *KRT75* (AUC = 0.819; Fig. 10i), *GFRA1* (AUC = 0.812; Fig. 10j), and *MMP3* (AUC = 0.709; Fig. 10k) also showed good diagnostic value.

### 3.9 Verification of hub genes in cSCC cell models

**Fig. 11:**
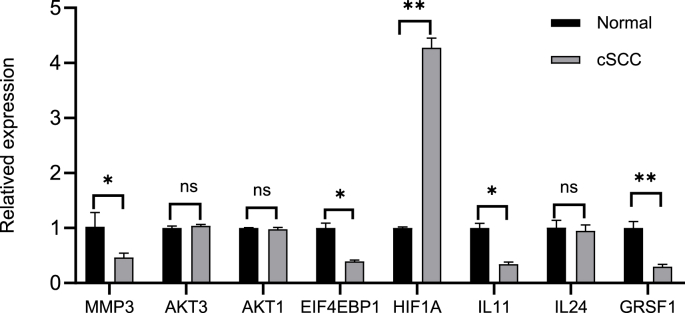
Validation the expression of hub genes. qPCR results for mRNA levels of the hub genes *MMP3, AKT3, AKT1, EIF4EBP1, HIF1A, IL11, IL24,* and *GRSF1*. Data are shown as mean ± SD. ∗p < 0.05, ∗∗p < 0.001, ns, no significance.

RT-qPCR in the cell lines showed that the mRNA expression of four hub genes (*MMP3, IL11, GRSF1,* and *EIF4EBP1*) was significantly decreased (P < 0.05) in cSCC cells compared to that of normal HaCat cells, whereas the mRNA expression of *HIF1A* was upregulated in cSCC cells compared to that of controls (P < 0.05) (Fig. 11). Notably, these findings were consistent with the expression patterns identified using the bioinformatics analysis.

## 4 Discussion

CSCC is a typical indolent tumor, with the majority of cases associated with a good prognosis. However, advanced, or metastatic cSCC has a higher incidence and mortality rate [2]. Hence, detecting potential biomarkers for the early diagnosis and treatment of patients with cSCC, aiming to reduce the chances of cSCC formation and spread, is crucial. Clinically, misdiagnosis based solely on the clinical presentations of cSCC occurs, with well-differentiated cSCC being commonly misdiagnosed as seborrheic keratosis, high-grade actinic keratosis, or keratoacanthoma. Poorly differentiated cSCC may be confused with amelanotic melanoma or cutaneous adnexal tumor and Merkel cell carcinoma [2]. Although dermoscopy is typically used for a differential diagnosis, clear dermoscopic diagnostic criteria are still lacking for cSCC [26]. Other non-invasive techniques currently lack sufficient evidence for routine diagnosis [27]. Although pathological biopsy is still considered the most reliable method for diagnosing cSCC [2]. Further molecular evidence is necessary to better understand the intricate biological features of cSCC and the challenging clinical presentations of atypical or refractory cases. Biomarkers play a crucial role in risk stratification and quantifying the likelihood of lesion progression, particularly in cases where early or precancerous lesions may not present clear clinical signs. Therefore, the development of a non-invasive diagnostic model is imperative to enhance early detection of cSCC.

The aberrant activation of the PI3K/AKT/mTOR pathway in cutaneous squamous cell carcinoma (cSCC) has been extensively investigated. Studies indicate that inhibitors targeting this pathway demonstrate significant therapeutic efficacy in cSCC. For example, LY3023414, a novel PI3K-AKT-mTOR inhibitor, has shown growth-suppressive effects on human cutaneous SCC cells in both in vitro and in vivo experiments [28]. Additionally, in both in vivo and in vitro experiments, AKT inhibitors suppress UV-induced cSCCby targeting the PI3K/AKT/mTOR signaling pathway [29]. However, in established tumors, monotherapy with PI3K/mTOR inhibitors may be insufficient, often requiring combination with other therapeutic strategies to enhance efficacy [30]. These findings collectively provide a solid theoretical foundation and preclinical evidence supporting the application of PI3K/AKT/mTOR pathway inhibitors in cSCC and its precursor lesions. Future research should prioritize exploring combination therapies involving these inhibitors to overcome drug resistance and improve treatment outcomes [31]. By further elucidating the mechanistic roles of the PI3K/AKT/mTOR pathway in cSCC pathogenesis, we anticipate the development of more effective treatment regimens to improve patient prognosis.

Through integrated analysis of GEO datasets and PI3K/AKT/mTOR pathway-related genes, we have systematically identified for the first time several key candidate genes—EIF4EBP1, GRSF1, IL11, HIF1A, and MMP3—several of which have not been systematically reported or validated in the context of cSCC. Notably, some genes had been reported in other tumor types but were not systematically investigated or validated in cSCC. The specific findings and implications are as follows: EIF4EBP1: While recognized as a key downstream regulator of the mTOR pathway and implicated in tumor growth in cancers such as breast and ewing sarcoma tumors [32,33], our study is the first to reveal its significant upregulation and potential diagnostic value in cSCC. GRSF1: While reported to promote tumor progression via the PI3K/AKT pathway in gastric and breast cancer [34,35].this research newly demonstrates its downregulation in cSCC and suggests a potential association with immune infiltration. IL11: Although belonging to the IL6 cytokine family and known for its pro-tumorigenic roles in lung and gastric cancers [36,37] our results represent the first report of its dysregulation in cSCC. HIF1A and MMP3: Previous studies have highlighted their roles in promoting invasion and metastasis in other squamous cell carcinomas or skin tumors [[38], [39], [40]], this study for the first time links both genes to the PI3K/AKT/mTOR signalling pathway and validates their expression changes via qPCR. These findings underscore the novelty of our study, as we link both MMP3 and HIF1A to the PI3K/AKT/mTOR signaling pathway and confirm their altered expression through qPCR experiments. We propose these genes as novel potential diagnostic biomarkers, thereby highlighting both the innovative contributions and cautious interpretation of our results.While the current findings elucidate molecular characteristics of confirmed cSCC, the performance of these differentially expressed genes (DEGs) in early or precancerous lesions—such as actinic keratosis or carcinoma in situ—requires further validation. Additionally, as this study has not yet performed protein-level validation in clinical skin biopsy specimens, further investigations using immunohistochemistry (IHC) or multiplex immunofluorescence (mIF) are essential to assess total and phosphorylated protein levels of PI3K/AKT/mTOR pathway components and evaluate their clinical diagnostic utility. In subsequent studies, we plan to incorporate transcriptomic data from precursor lesions to examine the continuity of molecular signatures and the early diagnostic potential of the identified DEGs. We also intend to collect pathologically confirmed skin biopsy samples to validate tissue-level expression differences of key molecules and further explore their translational value in clinical diagnostics.

The tumor microenvironment and its corresponding immune cells have a significant effect on controlling tumor progression and the response to treatment [41], especially immunotherapy [42]. Therefore, investigating the infiltration status of immune cells in cSCC is essential for exploring its tumor immune therapy mechanisms, predicting biomarkers, and identifying new therapeutic targets. By employing the CIBERSORT algorithm, we observed notable disparities in subsets of immune cells, including T lymphocytes, B lymphocytes, dendritic cells, natural killer cells, and myeloid cells, between the control and cSCC groups, aligning with earlier research discoveries [42,43]. The remarkable fluctuations in memory-quiescent CD4^+^ T lymphocytes, inexperienced CD4^+^ T lymphocytes, Tregs, and helper follicular T lymphocytes imply the active participation of T lymphocytes in cSCC. Significant differences in various subgroups or functional states of other immune cells also indicate their potentially pivotal roles in the immune mechanisms of cSCC. Prior studies have shown that tumor-infiltrating immune cells exhibit both common and specific features across different cancer types [43]. The diversity and functional status of tumor-infiltrating immune cells are crucial for immune therapy [43]. Consequently, conducting thorough investigations of immune cells that infiltrate tumors can enhance comprehension of the distinct mechanisms of tumor development and treatment responses, ultimately advancing the progress of personalized immune therapy approaches. This study thus provides new insight into the cSCC immune infiltration landscape and suggests predictive indicators for immune therapy. However, the CIBERSORT analysis in this study was primarily designed to investigate the overall distribution patterns of immune cells at a macroscopic level, rather than to precisely capture immune heterogeneity at single-cell resolution.In future work, we plan to leverage publicly available single-cell datasets or generate our own single-cell sequencing data to more deeply characterize the heterogeneity and functional states of immune cell populations within the cSCC tumor microenvironment.

This study provides a systematic analysis of the association between the PI3K/AKT/mTOR signaling pathway and cutaneous squamous cell carcinoma (cSCC). By integrating bioinformatic approaches with preliminary experimental validation, we have identified several hub genes within this pathway that may represent novel diagnostic biomarkers for cSCC. A clinically relevant diagnostic model was developed based on these findings, offering a foundation for further investigation into the molecular mechanisms underlying cSCC pathogenesis. However, it is important to emphasize that the current model is intended for research use rather than immediate clinical application. Several limitations should be acknowledged. First, as an exploratory investigation based on public databases, this study prioritized the identification of potentially significant genes rather than producing a clinically deployable prediction tool. Consequently, the model requires further validation in independent cohorts using RNA-Seq data or prospectively collected samples. Second, the absence of detailed clinical staging information in the included GEO datasets limited our ability to evaluate stage-specific expression patterns. Third, initial validation was conducted only at the mRNA level in cell lines; further studies involving human tissue samples and in vivo models are necessary to confirm the protein-level relevance and functional roles of these candidate genes. In summary, this work highlights the potential diagnostic value of PI3K/AKT/mTOR-related genes in cSCC and provides a framework for future research. Subsequent studies should focus on multi-level validation and clinical correlation to advance these findings toward translational applications.

## Ethics statement

Not Applicable.

## Funding

This work were supported by the Ningxia Natural Science Foundation of China (Grant No. 2024AAC03567) and the University-level Research Project of Ningxia Medical University (Grant No. XZ2024032).

## CRediT authorship contribution statement

**Bingyan Yang:** Writing – original draft. **Hongyang Zhang:** Visualization. **Lingdi Dong:** Data curation. **Jianjun Wang:** Formal analysis. **Nan Yu:** Writing – review & editing.

## Declaration of competing interest

The authors declare that there are no conflicts of interest regarding the publication of this paper.

## Data Availability

The data of this study are available in the Gene Expression Omnibus (https://www.ncbi.nlm.nih.gov/geo/query/acc.cgi?acc=GSE45164, https://www.ncbi.nlm.nih.gov/geo/query/acc.cgi?acc=GSE45216, https://www.ncbi.nlm.nih.gov/geo/query/acc.cgi?acc=GSE98767), the CIBERSORT (https://cibersort.stanford.edu/), the STRING database (http://string-db.org), and the ENCORI database (https://starbase.sysu.edu.cn/). The processed data and images used in the analysis are available in the Supplemental Material file.
