# Supplementary material for: Identification of PI3K/AKT/mTOR-related genes as diagnostic biomarkers for cutaneous squamous cell carcinoma
Source: Biochem Biophys Rep. 2025 Dec 2;45:102355. doi: 10.1016/j.bbrep.2025.102355 (PMC12719196; doi:10.1016/j.bbrep.2025.102355)
Supplement: Multimedia component 1 [file mmc1.pdf]

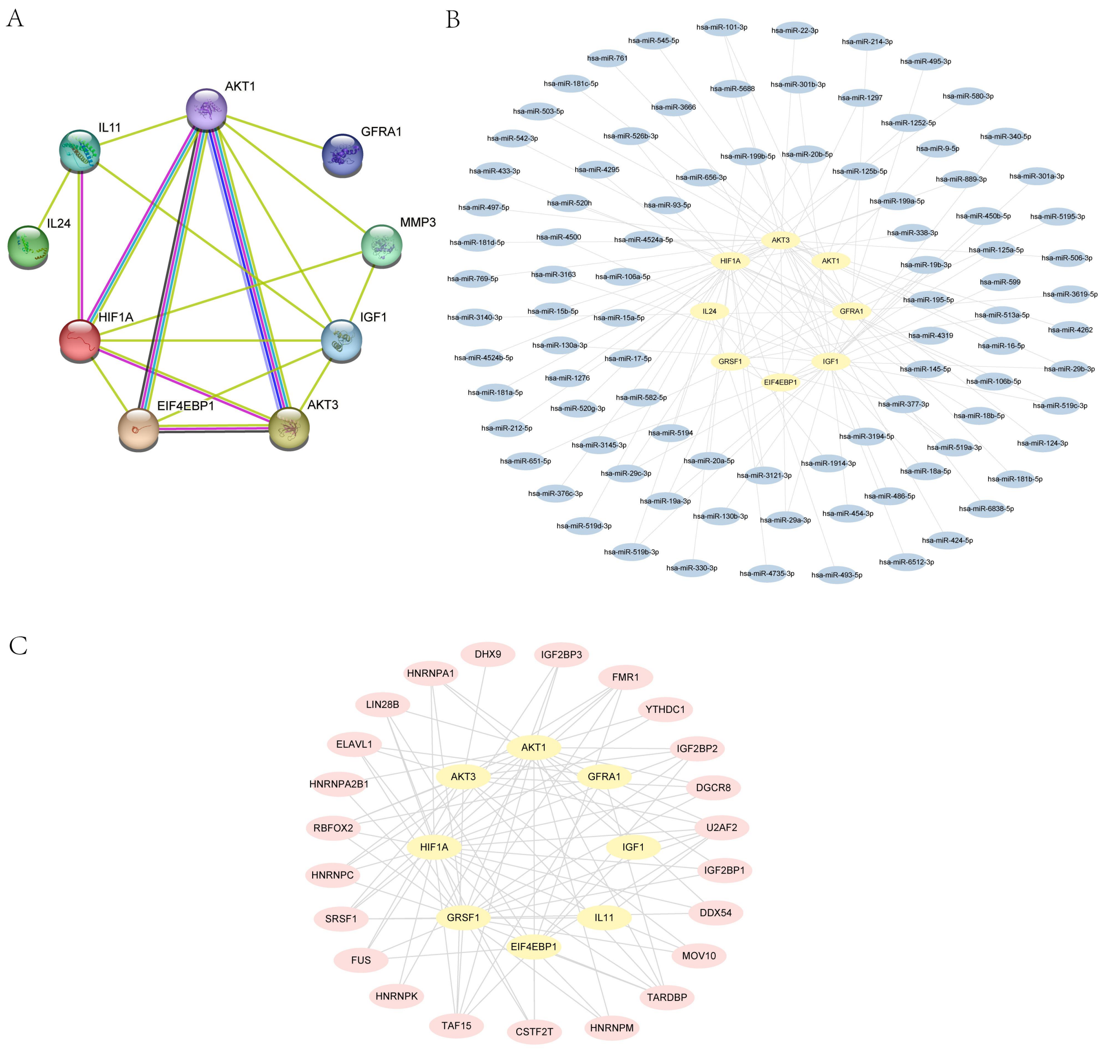

Figure 1S. Protein–protein, hub genes–miRNA, and hub genes-RBP interaction networks. (**a**) PPI network of PI3K/Akt/mTOR-RDEGs. (**b**) Hub genes–miRNA interaction network. (**c**) Hub genes–RBP interaction network. The yellow, blue, and pink ellipses represent mRNAs, miRNAs, and RBPs, respectively. PPI: protein–protein interaction; PI3K/Akt/mTOR-RDEGs: PI3K/Akt/mTOR-related differentially expressed genes; RBP: RNA-binding protein.
