# Supplementary material for: Identification of PI3K/AKT/mTOR-related genes as diagnostic biomarkers for cutaneous squamous cell carcinoma
Source: Biochem Biophys Rep. 2025 Dec 2;45:102355. doi: 10.1016/j.bbrep.2025.102355 (PMC12719196; doi:10.1016/j.bbrep.2025.102355)
Supplement: Multimedia component 2 [file mmc2.pdf]

Supplementary Table S1. PI3K/AKT/mTOR-related genes

Related genes
AKT1
AKT2
AKT3
BRAF
CAB39
CAB39L
DDIT4
EIF4B
EIF4E
EIF4E1B
EIF4E2
EIF4EBP1
HIF1A
IGF1
INS
MAPK1
MAPK3
MLST8
MTOR
PDPK1
PGF
PIK3CA
PIK3CB
PIK3CD
PIK3CG
PIK3R1
PIK3R2
PIK3R3
PIK3R5
PRKAA1
PRKAA2
RHEB
RICTOR
RPS6
RPS6KA1
RPS6KA2
RPS6KA3
RPS6KA6
RPS6KB1
RPS6KB2
RPTOR

STK11
STRADA
TSC1
TSC2
ULK1
ULK2
ULK3
VEGFA
VEGFB
VEGFC
VEGFD
NOX4
PHLDA2
LncIRS1
SIK2
MORC2
CTGF
SHP2
NLGN3
CMTM5
CYP7B1
UBE2T
UBE2J1
CYP2E1
KIFC1
LKB1
GATA5
Gtf2i
FAM83A
MRPS17
CD24
TOP2B
Rab11a
HOXA5
S100A4
UL46
PCDH10
Nectin-4
LRIG1
TRIM47
GRSF1
LRIG2
SIX4
TRIM37

KLHL22
GAS6
AKR1C2
SLCO4C1
PlGF
TET2
FAM83H
SLC34A2
Gtf2ird1
TIMP-4
BCHE
TYR
DCT
KRT222
RP11-507K12.1
COL6A5
PMP2
GFRA1
SCN7A
CDH19
IL11
OSM
MMP3
KRT75
MMP1
IL6
IL1B
IL24
SP7
ADGRG3
PTEN
AKT

Supplementary Table S2. The primers for qPCR

Primer name	Primer sequence(5’ -3’)	Product length(bp)	Annealing temperature(℃)
β-actin F	TGGCACCCAGCACAATGAA	186	58.0
β-actin R	CTAAGTCATAGTCCGCCTAGAAGCA		
AKT1 F	GCTATTGTGAAGGAGGGTTGG	305	59.3
AKT1 R	ACAGTCTGGATGGCGGTTG		
AKT3 F	TACCTTATCCCCTCAACAACTTTTC	160	58.7
AKT3 R	TCTGTCCATTCTTCCCTTTCCT		
MMP3 F	GCAGTTAGAGAACATGGAGACTTTT	259	58.6
MMP3 R	GGAACCGAGTCAGGTCTGTG		
EIF4EBP1 F	GAACTCACCTGTGACCAAAACAC	130	59.0
EIF4EBP1 R	CCGCTTATCTTCTGGGCTATT		
GRSF1 F	TGCTGATTTTGGAACTACGTCTTC	116	59.9
GRSF1 R	GATTCTAACAGGCTTGAGTGGAG		
HIF-1A F	GAAAGCAGTTCCGCAAGC	197	57.2
HIF-1A R	TTATGTATGTGGGTAGGAGATGG		
IL-11 F	GGGGACCACAACCTGGATT	119	60.2
IL-11 R	TGCCGCAGGTAGGACAGTAG		
IL-24 F	TGCAGAACGTCTCGGATGC	232	60.4
IL-24 R	CGGAATAGCAGAAACCGCCT		

qPCR:quantitative polymerase chain reaction

Supplementary TableS3. A total of 102 pairs of mRNA–miRNA interaction network nodes

**mRNA**	**miRNA**
AKT1	CSTF2T
AKT1	DDX54
AKT1	DGCR8
AKT1	FMR1
AKT1	HNRNPA1
AKT1	HNRNPA2B1
AKT1	HNRNPC
AKT1	HNRNPK
AKT1	HNRNPM
AKT1	IGF2BP1
AKT1	IGF2BP2
AKT1	IGF2BP3
AKT1	LIN28B
AKT1	RBFOX2
AKT1	SRSF1
AKT1	TAF15
AKT1	TARDBP
AKT1	U2AF2
AKT1	YTHDC1
AKT3	DGCR8
AKT3	DHX9
AKT3	ELAVL1
AKT3	FMR1
AKT3	FUS
AKT3	HNRNPC
AKT3	IGF2BP2
AKT3	MOV10
AKT3	RBFOX2
AKT3	SRSF1
AKT3	TAF15
AKT3	TARDBP
AKT3	U2AF2
EIF4EBP1	CSTF2T
EIF4EBP1	FMR1
EIF4EBP1	FUS
EIF4EBP1	IGF2BP2
EIF4EBP1	LIN28B
EIF4EBP1	TARDBP
EIF4EBP1	U2AF2
GFRA1	ELAVL1
GFRA1	RBFOX2

GFRA1	TAF15
GFRA1	TARDBP
GRSF1	CSTF2T
GRSF1	DDX54
GRSF1	DGCR8
GRSF1	ELAVL1
GRSF1	FMR1
GRSF1	FUS
GRSF1	HNRNPA1
GRSF1	HNRNPA2B1
GRSF1	HNRNPC
GRSF1	HNRNPM
GRSF1	IGF2BP1
GRSF1	IGF2BP2
GRSF1	IGF2BP3
GRSF1	LIN28B
GRSF1	MOV10
GRSF1	RBFOX2
GRSF1	SRSF1
GRSF1	TAF15
GRSF1	TARDBP
GRSF1	U2AF2
HIF1A	CSTF2T
HIF1A	DDX54
HIF1A	DGCR8
HIF1A	ELAVL1
HIF1A	FMR1
HIF1A	FUS
HIF1A	HNRNPA1
HIF1A	HNRNPC
HIF1A	HNRNPK
HIF1A	HNRNPM
HIF1A	IGF2BP1
HIF1A	IGF2BP2
HIF1A	IGF2BP3
HIF1A	LIN28B
HIF1A	MOV10
HIF1A	RBFOX2
HIF1A	SRSF1
HIF1A	TAF15
HIF1A	TARDBP
HIF1A	U2AF2
HIF1A	YTHDC1
IGF1	HNRNPA1

IGF1	TAF15
IGF1	U2AF2
IL11	SRSF1
IL11	U2AF2

miRNA:microRNA.

Supplementary TableS4. A total of 89 mRNA–RBP interaction network nodes

mRNA	RBP
AKT1	CSTF2T
AKT1	DDX54
AKT1	DGCR8
AKT1	FMR1
AKT1	HNRNPA1
AKT1	HNRNPA2B1
AKT1	HNRNPC
AKT1	HNRNPK
AKT1	HNRNPM
AKT1	IGF2BP1
AKT1	IGF2BP2
AKT1	IGF2BP3
AKT1	LIN28B
AKT1	RBFOX2
AKT1	SRSF1
AKT1	TAF15
AKT1	TARDBP
AKT1	U2AF2
AKT1	YTHDC1
AKT3	DGCR8
AKT3	DHX9
AKT3	ELAVL1
AKT3	FMR1
AKT3	FUS
AKT3	HNRNPC
AKT3	IGF2BP2
AKT3	MOV10
AKT3	RBFOX2
AKT3	SRSF1
AKT3	TAF15
AKT3	TARDBP
AKT3	U2AF2
EIF4EBP1	CSTF2T
EIF4EBP1	FMR1
EIF4EBP1	FUS
EIF4EBP1	IGF2BP2
EIF4EBP1	LIN28B
EIF4EBP1	TARDBP
EIF4EBP1	U2AF2
GFRA1	ELAVL1
GFRA1	RBFOX2

GFRA1	TAF15
GFRA1	TARDBP
GRSF1	CSTF2T
GRSF1	DDX54
GRSF1	DGCR8
GRSF1	ELAVL1
GRSF1	FMR1
GRSF1	FUS
GRSF1	HNRNPA1
GRSF1	HNRNPA2B1
GRSF1	HNRNPC
GRSF1	HNRNPM
GRSF1	IGF2BP1
GRSF1	IGF2BP2
GRSF1	IGF2BP3
GRSF1	LIN28B
GRSF1	MOV10
GRSF1	RBFOX2
GRSF1	SRSF1
GRSF1	TAF15
GRSF1	TARDBP
GRSF1	U2AF2
HIF1A	CSTF2T
HIF1A	DDX54
HIF1A	DGCR8
HIF1A	ELAVL1
HIF1A	FMR1
HIF1A	FUS
HIF1A	HNRNPA1
HIF1A	HNRNPC
HIF1A	HNRNPK
HIF1A	HNRNPM
HIF1A	IGF2BP1
HIF1A	IGF2BP2
HIF1A	IGF2BP3
HIF1A	LIN28B
HIF1A	MOV10
HIF1A	RBFOX2
HIF1A	SRSF1
HIF1A	TAF15
HIF1A	TARDBP
HIF1A	U2AF2
HIF1A	YTHDC1
IGF1	HNRNPA1

IGF1	TAF15
IGF1	U2AF2
IL11	SRSF1
IL11	U2AF2

RBP:RNA-binding protein
